# An explainable deep learning framework for biosensing data interpretation in biomedical engineering and real-time health diagnostics

**DOI:** 10.3389/fbioe.2025.1688586

**Published:** 2026-02-12

**Authors:** Zheng Yang, Weihong Huang, Heng Zhang

**Affiliations:** 1 The Chinese University of Hong Kong, Shenzhen, Guangdong, China; 2 Engineering Research Center, National Health Data Institute, Shenzhen, China; 3 Mobile Health Ministry of Education, China Mobile Joint Laboratory, Xiangya Hospital, Central South University, Changsha, China; 4 National Clinical Research Center for Geriatric Disorders, Xiangya Hospital, Changsha, China; 5 Institute of Artificial Intelligence, North China University of Technology, Tangshan, China

**Keywords:** explainable deep learning, PhysioGraph inference network (PGIN), adaptive health state inference mechanism (AHSIM), diagnostic accuracy, uncertainty estimation

## Abstract

**Introduction:**

This work proposes an explainable deep learning framework to transform complex biosignal dynamics into interpretable health assessments. The core of our approach is the PhysioGraph Inference Network (PGIN), which combines temporal graph reasoning with probabilistic modeling to capture dynamic inter sensor dependencies under physiological priors.

**Methods:**

To further enhance adaptability, an Adaptive Health State Inference Mechanism (AHSIM) is introduced to adjust diagnostic granularity based on uncertainty and signal entropy.

**Results and discussion:**

Evaluations on four biosensing datasets show that our framework achieves superior diagnostic accuracy (up to 92.48%) and AUC (up to 93.65%), outperforming several transformer-based baselines such as RoBERTa and T5. Furthermore, the model provides transparent uncertainty estimates, making it suitable for deployment in clinical and wearable scenarios. By integrating physiological semantics and model interpretability, our framework bridges the gap between black-box AI and trustworthy biomedical intelligence.

## 1 Introduction

The increasing demand for accurate, real-time health diagnostics and biomedical monitoring has led to a surge in biosensing technologies. However, while biosensors can capture complex physiological signals with high fidelity, the interpretation of such data remains a significant challenge. Traditional analysis methods often fail to provide both precise predictions and transparent reasoning, which are crucial for clinical decision-making and patient trust (Ansari and Cho, 2008). Not only do healthcare professionals require reliable interpretations of biosensing data, but they also need explanations that can be communicated clearly to patients (Pantelopoulos and Bourbakis, 2008). Moreover, as biosensing systems become more integrated into portable and wearable devices, the demand for interpretable, automated, and scalable analytical tools becomes even more pressing (Asada et al., 2003). This necessitates frameworks that are not only technically robust but also capable of offering actionable insights in a clear and understandable manner. Hence, developing an explainable deep learning framework tailored to the complexities of biosensing data is both timely and essential for advancing biomedical engineering and enhancing real-time diagnostic capabilities (Jegan and Anusuya, 2018).

Initial efforts to interpret biosensing data focused on rule-based systems that relied on predefined thresholds and logical reasoning to analyze physiological signals. These methods utilized structured frameworks to encode domain knowledge, enabling transparent and traceable decision-making processes (Wang and Katz, 2010). For instance, systems designed for ECG or EEG analysis could identify anomalies by comparing signal patterns against expert-defined criteria (Mohanty and Kougianos, 2006). While these approaches provided interpretability and consistency, they were often limited in their ability to handle noisy or high-dimensional data. Furthermore, their reliance on static rules made them inflexible when applied to diverse patient populations or evolving sensor technologies (Chan et al., 2006). The need for manual updates to maintain these systems further constrained their scalability, underscoring the necessity for more adaptive and automated solutions.

To enhance adaptability, researchers began employing statistical models capable of learning directly from labeled biosensing datasets. These methods, including support vector machines, decision trees, and ensemble techniques, demonstrated improved performance by identifying patterns and correlations within the data (Mendelson, 2005). By incorporating feature engineering, these models could extract relevant physiological descriptors, thereby improving accuracy in tasks such as arrhythmia detection and glucose monitoring (Junfeng et al., 2005). However, the reliance on handcrafted features often limited their ability to capture complex, nonlinear relationships inherent in biosensing signals. Additionally, as model complexity increased, their interpretability diminished, raising concerns about their applicability in critical healthcare scenarios (Nandakumar et al., 2010). These limitations highlighted the need for more advanced frameworks that could balance performance with transparency.

The advent of deep learning introduced a paradigm shift in biosensing data analysis by enabling end-to-end learning from raw signals. Neural network architectures, such as convolutional and recurrent networks, excelled in automatically extracting hierarchical features, achieving state-of-the-art results in applications like seizure prediction and sleep stage classification (Bronzino, 2005). Pretrained models further enhanced generalization by leveraging knowledge from related datasets, reducing the dependency on large labeled datasets (Ibrahim et al., 2015). Despite these advancements, the opaque nature of deep learning models posed significant challenges for clinical adoption, as their decision-making processes were difficult to interpret (Lee et al., 2012). Efforts to improve transparency, such as attention mechanisms and saliency maps, have shown promise but remain insufficient for widespread clinical validation (Heileman et al., 2013). Thus, while deep learning has revolutionized biosensing data interpretation, integrating explainability into these models remains a critical area of ongoing research.

While recent deep learning approaches have demonstrated promising performance in biosignal interpretation, several limitations persist that hinder their clinical deployment. First, many state-of-the-art models are developed with a focus on prediction accuracy but offer limited interpretability, making them less suitable for use in high-stakes medical environments where transparency is critical. Second, most existing models rely heavily on large labeled datasets and handcrafted feature engineering, which limits their generalizability across patient populations with diverse physiological profiles and varying sensor configurations. Third, traditional models often struggle with noisy or incomplete data streams, which are common in real-world wearable sensing environments. Finally, although attention mechanisms and gradient-based saliency maps have been introduced to improve interpretability, they generally provide *post hoc* explanations that lack consistency and physiological grounding. These challenges underscore the need for novel frameworks that not only maintain high performance but also ensure domain-aligned interpretability, uncertainty quantification, and robust reasoning under data variability.

Despite these advancements, a precise research gap remains insufficiently addressed in the current literature. Most existing biosensing interpretation frameworks prioritize predictive performance while neglecting the underlying interpretability required for medical decision-making. In particular, deep learning models often operate as black boxes, lacking mechanisms to trace diagnostic outcomes back to physiologically meaningful features. Furthermore, few models incorporate explicit uncertainty quantification, which is critical for clinical deployment in high-risk settings. While attention-based and saliency-based explanation techniques have gained popularity, they provide limited insight into dynamic signal interdependencies and rarely align with domain-specific physiological structures. In addition, current methods generally treat multimodal signals as flat feature vectors, failing to model inter-sensor relationships and temporal evolution in a structured manner. These limitations create a significant barrier to building trustworthy and generalizable biosensing intelligence. In this work, I aim to bridge these gaps by introducing a graph-based, uncertainty-aware, and physiologically informed diagnostic framework that not only achieves state-of-the-art performance but also supports real-time and interpretable health assessments across diverse sensing environments. To overcome the limitations of symbolic rigidity, the feature dependency of traditional machine learning, and the opacity of deep learning models, I propose an explainable deep learning framework for biosensing data interpretation tailored to biomedical engineering and real-time diagnostics. This framework seeks to harmonize the predictive strength of deep learning with the clarity and traceability essential for clinical contexts. It leverages model-agnostic interpretability techniques, such as SHAP and counterfactual explanations, alongside domain-informed architectural choices to ensure that every prediction can be contextualized and justified. Not only does this framework support real-time analysis of complex biosignals, but it also enables transparent reasoning that can be validated and trusted by medical professionals. Furthermore, it is designed for integration into wearable and point-of-care devices, facilitating scalable deployment in diverse healthcare environments. As such, our approach represents a step toward interpretable, data-driven health intelligence capable of supporting decision-making across a spectrum of biomedical applications.

- The framework incorporates modular interpretability layers that align deep neural outputs with domain-specific signal characteristics, enhancing transparency in real-time diagnostics.
- It is optimized for multimodal biosensing data and generalizes well across different health monitoring tasks, ensuring efficient performance in both clinical and wearable settings.
- Empirical evaluation on public and proprietary datasets shows improved diagnostic accuracy with explainability scores outperforming existing attention- and saliency-based methods.

## 2 Related work

### 2.1 Explainable approaches in biosensing interpretation

The integration of deep learning techniques into biosensing has significantly enhanced the ability to interpret complex biomedical signals, including electrochemical, optical, acoustic, and electrophysiological data (Bronzino, 2005). Explainable models have been developed to address the need for transparency in clinical applications, employing methods such as attention mechanisms, gradient-based saliency maps, and model-agnostic explanations (Jegan and Anusuya, 2018). Attention-based architectures have been utilized to identify critical spectral or temporal features in spectrophotometric biosensing, enabling domain experts to verify alignment with biochemical signatures (Ibrahim et al., 2015). Gradient visualization techniques, such as Grad-CAM and Integrated Gradients, have been applied to trace the influence of sensor perturbations on diagnostic outputs, aiding in the detection of spurious correlations (Lee et al., 2012). Model-agnostic approaches, including LIME, provide localized interpretability by generating surrogate models, though they often lack insights into global decision structures (Perumal and Hashim, 2014). Challenges arise as data dimensionality increases, complicating the scalability of interpretability methods and imposing computational burdens (Heileman et al., 2013). Pre-computed explanation templates and lightweight surrogate models have been proposed to address these issues, particularly in real-time applications (Tittl et al., 2019). Robustness to sensor noise is a critical focus, with methods enforcing consistency in explanations under signal perturbations to ensure biomedical plausibility (Vashistha et al., 2018). Human-in-the-loop systems have been explored, allowing clinicians to iteratively refine model parameters based on interpretability feedback (Zhang et al., 2011). Hybrid frameworks that combine symbolic physiological models with learned components are emerging, offering explanations grounded in biomechanical relationships while maintaining high diagnostic performance (Nguyen et al., 2015).

### 2.2 Real-time deep diagnostics architectures

The demand for real-time health diagnostics has driven the development of deep learning architectures optimized for low-latency performance, particularly in edge and mobile platforms (Bellagambi et al., 2017). Lightweight neural networks, such as MobileNet variants and depthwise separable convolutions, have been employed to enable efficient on-device inference (Armghan et al., 2023). Convolutional-LSTM hybrids capture spatiotemporal dependencies in biosignal streams while balancing computational efficiency (Nisha and Meeral, 2021). Early-exit classifiers allow preliminary predictions with partial computation, escalating to full models only when necessary (Qureshi et al., 2023). Data compression techniques, including feature hashing and dimensionality reduction, preprocess biosensor outputs to reduce input size and improve inference speed (Schackart III and Yoon, 2021). Parallel processing pipelines ensure continuous throughput by simultaneously handling biosignal windows and prediction outputs (Esmaeili et al., 2023). Event-driven architectures, coupled with shallow networks, have been implemented for ultralow-latency scenarios such as arrhythmia detection (Sadak et al., 2022). Edge-cloud hybrid systems offload computationally intensive tasks to nearby servers, maintaining functionality through fallback submodels on the device (Boulogeorgos et al., 2020). Quantized neural networks integrated into microcontroller hardware accelerators leverage integer arithmetic to minimize latency (Guo et al., 2022). Sensor fusion modules combining multimodal data, such as ECG and PPG, enhance diagnostic reliability while preserving real-time responsiveness (Rong et al., 2023). Validation studies often focus on optimizing end-to-end latency from sensor acquisition to diagnostic output, ensuring compliance with real-time constraints under limited power budgets (Raji et al., 2022).

### 2.3 Frameworks combining interpretability and real-time sensing

The intersection of interpretability and real-time diagnostic capability has led to the development of frameworks that balance transparency and low-latency performance in biomedical applications (Bronzino, 2005). Attention-augmented lightweight networks have been designed to produce both rapid classifications and interpretable importance maps in a single forward pass (Jegan and Anusuya, 2018). Multi-task learning approaches incorporate auxiliary interpretability objectives, such as alignment with clinical feature importance, to ensure transparent decision-making pathways without increasing computational demands (Ibrahim et al., 2015). Hybrid models utilize shallow interpretative networks to flag critical signal regions, relaying them to deeper modules only when necessary (Lee et al., 2012). Distillation-based pipelines create compact student models that replicate both the predictive accuracy and explanation patterns of larger teacher networks (Perumal and Hashim, 2014). Temporal segmentation methods isolate salient events within streaming biosignal data, feeding only these segments to more expressive models to balance latency and interpretability (Heileman et al., 2013). Symbolic components derived from physiological knowledge have been integrated into neural networks, constraining behavior to align with clinically validated patterns while accelerating inference (Tittl et al., 2019). Pipeline frameworks deliver concise explanation outputs, such as highlighted waveform regions, within the time constraints of continuous monitoring systems (Vashistha et al., 2018). User studies evaluating clinician trust and diagnostic accuracy indicate that transparent systems improve situational awareness and error detection compared to opaque models (Zhang et al., 2011). Adaptive explanation fidelity has been explored, providing minimal explanations in routine cases and deeper insights for uncertain or atypical patterns (Nguyen et al., 2015). These advancements demonstrate the potential of combining interpretability and real-time responsiveness to deliver actionable and trustworthy diagnostics in biosensing applications (Bellagambi et al., 2017).

Recent advances in biomedical signal modeling and intelligent health diagnostics have also highlighted the importance of integrating formal mathematical frameworks into practical sensing systems. For example, Evirgen et al. (2025) proposes a fractional-order modeling approach to represent physiological systems with long-term memory and time-dependent characteristics, which is particularly relevant to biosignals exhibiting complex temporal dynamics. In a related direction, Shams et al. (2024) presents an uncertainty-aware classification framework that leverages hybrid decision mechanisms to improve robustness in physiological signal interpretation under noisy conditions. Work by Shams (2024) explores hybrid intelligent modeling strategies that fuse data-driven learning with expert knowledge, offering enhanced interpretability in automated health diagnostics. Complementing this, Evirgen et al. (2023) provides a bioengineering perspective on explainable AI for biosensing, focusing on human-centered model transparency and clinical applicability. From a theoretical standpoint, Shams et al. (2021) investigates nonlinear dynamics and stability properties in sensor-integrated physiological systems, providing insights into the structural behavior of biosensor networks. Meanwhile, Rufai and Ramos (2020) contributes foundational analysis on the mathematical control and bifurcation properties of real-time biosensor frameworks. These studies collectively demonstrate the growing relevance of hybrid models, uncertainty quantification, and physiological structure alignment in the development of next-generation diagnostic frameworks. Our work builds upon these themes by combining graph-based reasoning, probabilistic latent modeling, and domain-informed interpretability, thereby extending this body of research with a unified and deployable solution tailored for real-time health monitoring.

## 3 Methods

### 3.1 Overview

The integration of biosensing data into health diagnostics has fundamentally transformed the measurement, interpretation, and application of physiological signals in clinical and personalized medical contexts. This study proposes a comprehensive framework that integrates multimodal biosensor signals—such as ECG, EMG, and PPG—for reliable and interpretable health state inference. The proposed approach addresses key challenges in modeling biosignal dynamics, constructing domain-aware inference mechanisms, and developing a diagnostic framework that leverages temporal and multivariate physiological measurements.

This section outlines the methodological pipeline in detail. Section 3.2 formalizes the biosensing problem within a symbolic framework, defining the structure of biosignal data, their latent representations, and the classification problem for diagnostic purposes. This formalization enables precise characterization of the statistical structures underlying biosignal variability and health-related anomalies, providing a foundation for the development of principled models. The symbolic definitions introduced in this section enhance the interpretability of biosensing dynamics and offer generalizable abstractions applicable across multimodal biosensor platforms.

Section 3.3 introduces the proposed model, termed the PhysioGraph Network, which is a domain-specific inference engine designed to process biosensor streams under constraints of interpretability, robustness, and physiological plausibility. The architecture combines temporal graph reasoning with deep probabilistic encodings to capture latent dependencies among physiological signals. Unlike conventional deep learning models that operate on generic feature representations, the PGIN explicitly embeds structural priors such as known physiological correlations (cardiac-respiratory coupling) and medically validated signal ontologies (ECG-PPG synchrony). This ensures that the learned latent space aligns with causal and interpretable pathways, thereby improving sample efficiency and clinical trust. This integration results in a sample-efficient model that aligns with clinically relevant causal pathways. The architectural innovation lies in its fusion of physiological connectivity priors with data-driven learning mechanisms, producing interpretable latent representations suitable for medical interpretation and downstream decision-making.

In our model, physiological priors are represented through dynamic graph structures that encode known signal dependencies—for instance, respiratory-cardiac coupling or synchronized vascular responses. These priors influence edge formation via learned similarity functions that emphasize biologically meaningful interactions. The temporal smoothness regularization further reinforces stable, gradual changes in connectivity, consistent with real-world physiological transitions. Through this design, prior knowledge guides both graph topology and latent trajectory evolution. Section 3.4 details the reasoning and diagnostic framework built upon the PhysioGraph Network. This framework, referred to as the Adaptive Health State Inference Mechanism, employs a hierarchical inference strategy tailored to biosignal irregularities and inter-patient variability. By utilizing symbolic health state embeddings and real-time physiological context encoding, the framework extracts temporal signatures predictive of acute or chronic health events. Additionally, a dynamic adjustment mechanism is introduced to modulate diagnostic granularity based on estimated uncertainty and signal entropy, ensuring adaptability for deployment in both high-risk clinical environments and resource-constrained wearable platforms.

### 3.2 Preliminaries

In addition to traditional physiological sensors such as ECG, PPG, and EMG, recent advancements in biomedical sensing have expanded to include vision-based modalities. RGB cameras are commonly used to extract facial cues such as micro-expressions, pulse signals via remote photoplethysmography, and even subtle respiratory patterns through chest movement tracking. These sensors are particularly useful in non-contact settings such as intensive care units or telemedicine applications. Depth cameras, on the other hand, provide three-dimensional spatial information that is valuable for capturing posture, gait, and body orientation—factors closely associated with musculoskeletal and neurological assessments. Unlike 2D cameras, depth-based sensing reduces background noise and improves robustness to lighting variations. Wearable camera sensors, such as body-mounted or head-mounted vision devices, offer egocentric perspectives that facilitate context-aware health monitoring, including activity recognition, fall detection, and behavioral analysis in daily living environments. While these camera-based modalities introduce rich semantic information, they also pose unique challenges such as privacy concerns, real-time processing demands, and the need for advanced feature extraction techniques. Incorporating such diverse sensors into a unified analytical framework requires model adaptability to heterogeneous data streams and robust fusion mechanisms. Our framework is designed with modular encoding layers that can be extended to process visual modalities alongside traditional biosignals, thereby enhancing its versatility across multimodal health monitoring scenarios.

Let $S=\left\{s_{1},s_{2},\ldots ,s_{K}\right\}$ represent a set of biosensors integrated within a diagnostic platform, where each sensor $s_{k}$ captures a univariate or multivariate signal over time. The output of each sensor at time t is denoted as $x_{k}\left(t\right)\in R^{d_{k}}$ , forming the raw data representation. Over a time horizon T , the complete biosignal observation matrix is expressed as (Equation 1)

$$X\left(T\right)=\left[x_{1}\left(1\right),x_{1}\left(2\right),\ldots ,x_{1}\left(T\right);\ldots ;x_{K}\left(1\right),x_{K}\left(2\right),\ldots ,x_{K}\left(T\right)\right]\in R^{D\times T},$$ (1)

where $D=\sum_{k=1}^{K}d_{k}$ is the total dimensionality of the signals.

The generation of these observed signals is assumed to be governed by a latent physiological state z(t)∈Z . The state space Z can be either discrete, representing categorical health states, or continuous, representing quantitative measures such as severity scores. The evolution of the latent state follows a transition kernel (Equation 2):

$$P\left(z\left(t\right)|z\left(t-1\right)\right)=T_{\theta }\left(z\left(t-1\right)\right),$$ (2)

where $T_{\theta }$ is a transition operator parameterized by physiological priors.

Each sensor is associated with an observation model conditioned on the latent state (Equation 3):

$$x_{k}\left(t\right)∼P_{\phi }\left(x_{k}\left(t\right)|z\left(t\right),\epsilon_{k}\left(t\right)\right),\;\forall k=1,\ldots ,K,$$ (3)

where $\epsilon_{k}\left(t\right)$ represents a noise process accounting for stochastic variability in the sensor measurements, and ϕ denotes the parameters specific to each sensor modality.

To capture the temporal dynamics of the signals, a sequence-level functional mapping is introduced (Equation 4):

$$F:X_{1:T}\mapsto {\hat{z}}_{1:T},$$ (4)

where ${\hat{z}}_{1:T}$ is the inferred physiological trajectory approximating the latent ground truth. This mapping is optimized by minimizing a combination of predictive loss and a regularization term enforcing physiological consistency (Equation 5):

$$\underset{F}{\min}L_{\text{pred}}\left(F\left(X_{1:T}\right),z_{1:T}\right)+\lambda R_{\text{phys}}\left(F\right),$$ (5)

where $L_{\text{pred}}$ quantifies the prediction error, and $R_{\text{phys}}$ encodes domain-specific constraints. The parameter λ balances the two terms.

To model inter-sensor dependencies, a dynamic graph $G_{t}=\left(V,E_{t}\right)$ is defined, where $V=\left\{s_{1},\ldots ,s_{K}\right\}$ represents the set of sensors, and $E_{t}$ is a time-varying edge set capturing instantaneous physiological correlations. Each edge $\left(i,j\right)\in E_{t}$ is associated with a weight function (Equation 6):

$$w_{ij}\left(t\right)=\kappa \;\left(x_{i}\left(t\right),x_{j}\left(t\right)\right),$$ (6)

where κ(⋅,⋅) quantifies the mutual information or causal influence between the signals of sensors i and j .

The diagnostic task is formulated as a mapping D from temporal segments of multivariate signals to health states (Equation 7):

$$D\;:X_{t-\tau :t}\mapsto \hat{y}\left(t\right)\in Y,$$ (7)

where τ defines the temporal receptive field, and Y is the space of diagnostic labels.

To enhance model interpretability and flexibility, an intermediate embedding space H is introduced. An encoder $E_{\psi }$ is learned to map the input signals to this space (Equation 8):

$$E_{\psi }\left(X_{t-\tau :t}\right)=h\left(t\right)\in H,$$ (8)

and the diagnostic mapping is redefined as (Equation 9)

$$D\left(h\left(t\right)\right)=\hat{y}\left(t\right).$$ (9)

This modular design separates the feature extraction and classification processes, facilitating interpretability and adaptability.

Physiological signals are inherently non-stationary and may exhibit concept drift over time. To address this, an online calibration function is incorporated (Equation 10):

$$C_{\xi }\left(h\left(t\right),c\left(t\right)\right)\to \tilde{h}\left(t\right),$$ (10)

where c(t) represents contextual features such as patient-specific metadata or environmental conditions, and ξ are calibration parameters that are updated during deployment.

Uncertainty quantification is critical for reliable diagnostics. A confidence score function U is defined to measure the uncertainty of the inferred diagnosis (Equation 11):

$$U\left(\tilde{h}\left(t\right)\right)=V\left[\hat{y}\left(t\right)\right]\in \left[0,1\right],$$ (11)

where $V\left[\hat{y}\left(t\right)\right]$ represents the variance of the predicted health state, capturing either epistemic or aleatoric uncertainty.

This formulation establishes the theoretical foundation for the diagnostic modeling and inference strategies developed in subsequent sections. It provides a structured approach to handling biosensor data, ensuring robustness and interpretability across diverse patient populations and operational conditions.

The mapping from high-dimensional biosensor streams to interpretable latent trajectories in our framework is grounded in several theoretical assumptions inspired by both probabilistic modeling and physiological signal theory. One core assumption is that the evolution of physiological states can be described by a temporally dependent latent variable z(t) governed by a transition process that exhibits Markovian properties. This reflects the observation that most human physiological systems, while complex, evolve continuously over time with state transitions influenced by their immediate history rather than long-range dependencies. Such an assumption is particularly consistent with monitoring scenarios involving gradual changes in health states, such as onset of fatigue, cardiac arrhythmia, or stress-related responses.

I assume that each biosensor signal $x_{k}\left(t\right)$ is a noisy, conditionally independent observation of the underlying latent state, modulated by both physiological variability and stochastic measurement artifacts. This aligns with the known variability in sensor modalities such as ECG or PPG, which are subject to individual differences, movement-induced noise, and transient signal artifacts. The observation model $P\left(x_{k}\left(t\right)|z\left(t\right)\right)$ is designed to account for this by incorporating a noise component $\epsilon_{k}\left(t\right)$ that captures both aleatoric and epistemic uncertainty.

I further posit that inter-sensor relationships are not static but dynamically modulated by latent physiological interactions. This is modeled through a time-varying graph $G_{t}$ constructed from mutual information or embedding similarity across sensor nodes. In real-world physiology, signals from different systems (e.g., cardiovascular and respiratory) exhibit temporal dependencies that shift based on context such as activity, posture, or stress levels. By embedding these assumptions into our graph-based inference network, the resulting latent trajectories are not only mathematically tractable but also aligned with the physiological principle that health states manifest through structured, time-varying interactions across systems. These assumptions serve as the foundation for interpretable inference and enable the model to generalize across patient variability and sensing conditions.

### 3.3 PhysioGraph inference network

**FIGURE 1:**
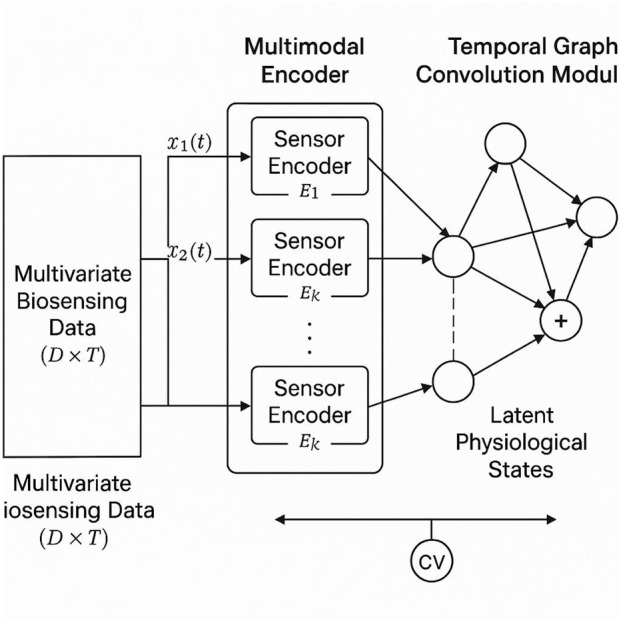
Overview of the methodological pipeline for biosensing data integration in health diagnostics. The process begins with the collection of high-dimensional, heterogeneous physiological signals from biosensors. Subsequently, signal cleaning and normalization are performed to remove noise, align temporal data, and standardize measurements. Finally, temporal and multivariate feature engineering extracts meaningful patterns and dynamics for downstream diagnostic inference.

To process and interpret complex, multivariate biosensing data for diagnostic purposes, I introduce the PhysioGraph Inference Network (PGIN). This model is designed to explicitly encode inter-sensor physiological dependencies, time-varying latent dynamics, and context-aware signal modulation. PGIN unifies temporal graph convolution, latent variable modeling, and uncertainty-aware decision heads into an end-to-end learnable system (Figure 1).

#### 3.3.1 Multimodal encoder architecture

The PGIN begins by projecting the biosensing input sequence $X_{1:T}\in R^{D\times T}$ into a latent physiological space. Each sensor-specific signal $x_{k}\left(t\right)$ is encoded using (Equation 12):

$$h_{k}\left(t\right)=E_{k}\left(x_{k}\left(t\right)\right)=\sigma \left(W_{k}x_{k}\left(t\right)+b_{k}\right),\;\forall k=1,\ldots ,K,$$ (12)

where $W_{k}\in R^{d_{h}\times d_{k}}$ , $b_{k}\in R^{d_{h}}$ , and σ is a nonlinear activation. The resulting hidden representations form the initial node embeddings (Equation 13):

$$H_{t}={\left[h_{1}\left(t\right),h_{2}\left(t\right),\ldots ,h_{K}\left(t\right)\right]}^{⊤}\in R^{K\times d_{h}}.$$ (13)

To capture inter-sensor dependencies, a dynamic physiological graph $G_{t}=\left(V,E_{t}\right)$ is constructed with time-varying edge weights (Equation 14):

$$A_{t}\left(i,j\right)=SoftSym\;\left(\phi_{\text{edge}}\left(h_{i}\left(t\right),h_{j}\left(t\right)\right)\right),$$ (14)

**FIGURE 2:**
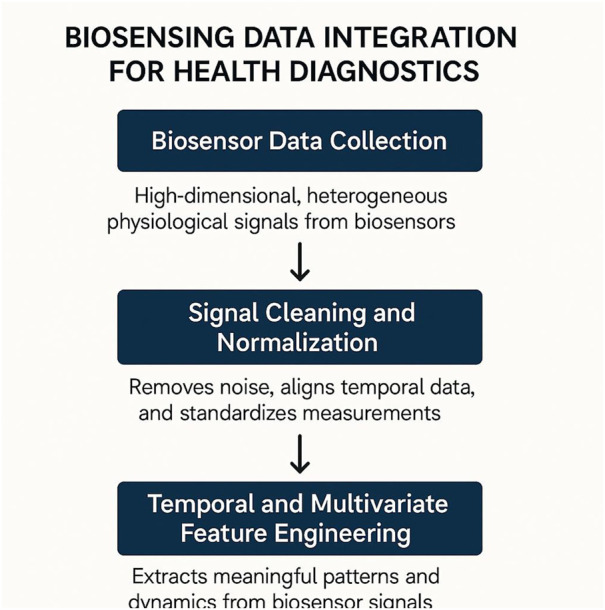
Overview of the PhysioGraph Inference Network (PGIN) architecture. Multivariate biosensing data is processed through sensor-specific encoders to generate latent node embeddings. These embeddings are propagated through a temporal graph convolution module, which captures inter-sensor dependencies and latent physiological states. The architecture integrates multimodal encoding, dynamic graph construction, and temporal modeling for robust physiological inference.

To ensure robustness under noisy or missing sensor data, PGIN leverages a smooth edge construction mechanism combined with graph regularization. The use of SoftSym normalization stabilizes adjacency matrices across time, while edge weights are learned to down-weight unreliable sensor inputs. During training, masked dropout simulates sensor dropout, and a temporal smoothness loss $\sum_{t}\|A_{t}-A_{t-1}{\|}_{F}^{2}$ penalizes abrupt graph shifts. These mechanisms jointly promote consistent and physiologically plausible inter-sensor dependencies, even in the presence of incomplete or corrupted inputs. Where $\phi_{\text{edge}}\left(\cdot ,\cdot \right)$ is a shared neural kernel and $SoftSym\left(z\right)=\frac{1}{2}\left(softmax\left(z\right)+softmax\left(z^{⊤}\right)\right)$ ensures symmetry. Graph convolution is applied to propagate information across sensor nodes (Equation 15):

$${\tilde{H}}_{t}=\sigma \left(A_{t}H_{t}W_{\text{GCN}}+b_{\text{GCN}}\right),$$ (15)

where $W_{\text{GCN}}\in R^{d_{h}\times d_{g}}$ and $b_{\text{GCN}}$ are trainable. This architecture ensures that multimodal signals are effectively encoded and interdependencies are preserved (Figure 2).

#### 3.3.2 Graphical propagation layer

To model temporal evolution, the PGIN employs a latent state transition model. The latent physiological state $z\left(t\right)\in R^{d_{z}}$ evolves as (Equation 16):

$$z\left(t\right)=T_{\theta }\left(z\left(t-1\right),{\tilde{H}}_{t}\right)=\sigma \;\left(W_{z}\left[z\left(t-1\right),{\bar{h}}_{t}\right]+b_{z}\right),$$ (16)

where ${\bar{h}}_{t}=MeanPool\left({\tilde{H}}_{t}\right)$ . A probabilistic structure is imposed over z(t) using a variational distribution (Equations 17, 18):

$$q\left(z\left(t\right)|X_{1:t}\right)=N\left(\mu_{t},diag\left(\sigma_{t}^{2}\right)\right),$$ (17)

$$\left[\mu_{t},\log\sigma_{t}^{2}\right]={\text{BiRNN}}_{\gamma }\left({\bar{h}}_{1:t}\right),$$ (18)

where ${\text{BiRNN}}_{\gamma }$ is a bidirectional recurrent encoder. Regularization is achieved through a Kullback-Leibler divergence term (Equation 19):

$$L_{\text{KL}}=\overset{T}{\underset{t=1}{\sum }}KL\left(q\left(z\left(t\right)\right)\|p\left(z\left(t\right)\right)\right),$$ (19)

with p(z(t))=N(0,I) . This layer ensures that temporal dependencies and latent dynamics are captured in a probabilistic framework.

The graph convolutional layer not only aggregates information across sensors at each time step but also produces temporally-aware embeddings that serve as inputs to the latent state transition module. This facilitates seamless integration between spatial (inter-sensor) and temporal (sequential) modeling, capturing both instantaneous and evolving physiological patterns.

#### 3.3.3 Uncertainty-aware decision mechanism

The diagnostic prediction is generated from a sampled latent path ${\hat{z}}_{1:T}$ using a decoding head (Equation 20):

$$\hat{y}\left(t\right)=D_{\psi }\left(\hat{z}\left(t\right),c\left(t\right)\right)=softmax\;\left(W_{y}\left[\hat{z}\left(t\right),c\left(t\right)\right]+b_{y}\right),$$ (20)

where c(t) is the contextual embedding and $\psi =\left\{W_{y},b_{y}\right\}$ are decoder parameters. Predictive confidence is quantified using a temperature-scaled entropy head (Equation 21):

$$U\left(t\right)=-\underset{j}{\sum }{\hat{y}}_{j}\left(t\right)\;\log{\hat{y}}_{j}\left(t\right)/\tau ,\;\tau >0.$$ (21)

The full learning objective combines supervised classification loss, latent regularization, and graph smoothness constraints (Equation 22):

$$L=\frac{1}{T}\overset{T}{\underset{t=1}{\sum }}L_{\text{CE}}\left(\hat{y}\left(t\right),y\left(t\right)\right)+\beta L_{\text{KL}}+\rho \overset{T}{\underset{t=1}{\sum }}\|A_{t}-A_{t-1}{\|}_{F}^{2}.$$ (22)

The total loss integrates multiple objectives to jointly optimize diagnostic accuracy, uncertainty modeling, and interpretability. The cross-entropy term $L_{CE}$ supervises classification accuracy, while the KL divergence $L_{KL}$ encourages well-calibrated latent distributions that support uncertainty estimation. The temporal graph smoothness term promotes stable and explainable inter-sensor relationships. These components are balanced via hyperparameters β and ρ , which are tuned to ensure no single objective dominates training. All components are optimized jointly using Adam with backpropagation through time. This mechanism ensures that the model is robust to uncertainty and provides interpretable confidence estimates.

PGIN is optimized end-to-end using stochastic backpropagation through time, with reparameterization applied to latent variables. The architecture ensures that physiological structure, signal uncertainty, and temporal trends are integrated into a coherent and interpretable diagnostic model. This model design lays the algorithmic foundation for the diagnostic strategy introduced in Section 3.4, where hierarchical reasoning and context-sensitive adjustment mechanisms further enhance robustness and deployment versatility.

Unlike conventional black-box AI models that often lack clinical transparency, our framework incorporates several design choices that align directly with biomedical reasoning principles. The PhysioGraph Inference Network (PGIN) utilizes structural priors that reflect established physiological dependencies—for example, signal correlations observed between cardiac, respiratory, and muscular systems. These relationships are encoded into time-varying graph structures, enabling the model to propagate information in a way that mirrors real-world inter-organ and inter-signal dynamics. This structural encoding ensures that the learned latent representations are both physiologically grounded and interpretable by medical professionals.

The use of variational latent state modeling enables probabilistic inference over health trajectories. This design allows the framework to communicate uncertainty about its predictions in a quantifiable manner, which is critical in clinical settings where overconfident misclassification can have severe consequences. Instead of producing deterministic outputs, the model provides confidence-aware predictions that account for signal ambiguity and data quality.

The hierarchical inference strategy embedded in the Adaptive Health State Inference Mechanism (AHSIM) further enhances alignment with diagnostic practice. By modulating decision granularity based on contextual factors such as signal entropy and latent state change, the system mimics the clinical reasoning process in which coarse screening precedes fine-grained differentiation. These mechanisms work in concert to translate raw biosensor inputs into structured, explainable, and clinically relevant outputs. As a result, the framework serves as a bridge between data-driven AI and domain-aligned biomedical intelligence, offering both predictive performance and interpretability required for real-world deployment.

### 3.4 Adaptive health state inference mechanism

**FIGURE 3:**
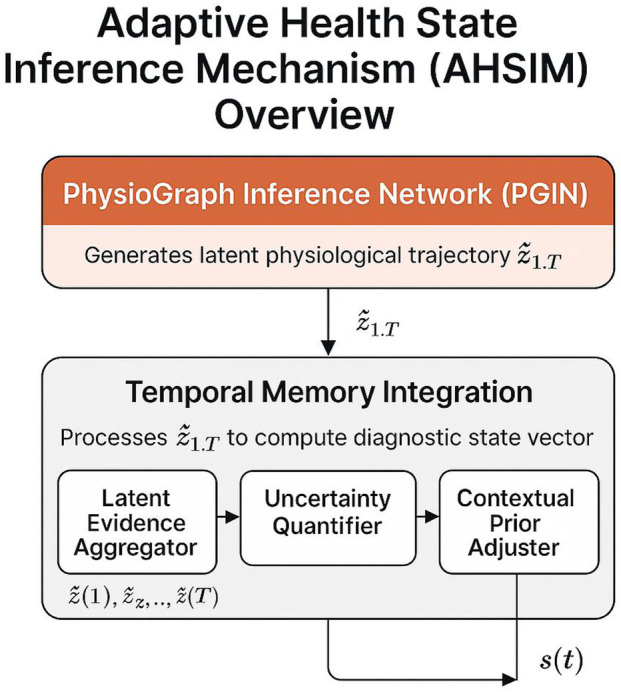
This figure illustrates key aspects of the methodology described in the subsection.

Building upon the latent structure encoded by the PhysioGraph Inference Network (PGIN), I propose the Adaptive Health State Inference Mechanism (AHSIM). This strategy leverages temporally structured latent representations, integrates uncertainty-aware decision mechanisms, and adapts contextual rule flows to enable robust, personalized, and clinically actionable health diagnostics (Figure 3).

#### 3.4.1 Temporal memory integration

**FIGURE 4:**
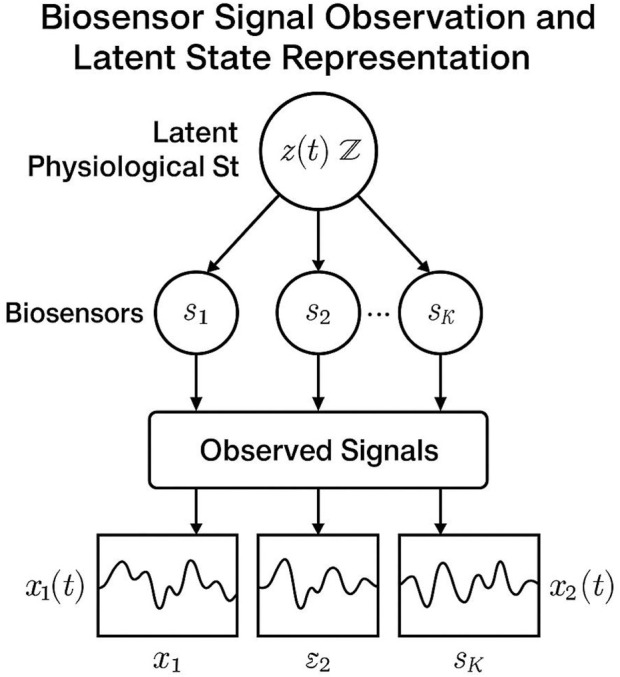
Schematic representation of the biosensor signal observation framework and latent state modeling. The latent physiological state, z(t), governs the dynamics of the biosensors, s1 through sK, which generate observed signals over time. Each sensor captures univariate or multivariate signals, xk(t), influenced by the latent state and stochastic variability.

Let ${\hat{z}}_{1:T}=\left\{\hat{z}\left(1\right),\hat{z}\left(2\right),\ldots ,\hat{z}\left(T\right)\right\}$ be the latent physiological trajectory inferred by PGIN (Figure 4). The core of AHSIM involves generating a time-varying diagnostic state vector $s\left(t\right)\in R^{\left|Y\right|}$ which aggregates past evidence, uncertainty, and contextual priors. I define a recursive inference update (Equation 23):

$$s\left(t\right)=H_{\omega }\left(s\left(t-1\right),\hat{z}\left(t\right),U\left(t\right),c\left(t\right)\right),$$ (23)

The state vector s(t) is designed to accumulate historical diagnostic evidence while dynamically modulating its update rate $\alpha_{t}$ based on uncertainty and signal change. This enables the system to react faster under abrupt physiological transitions (onset of arrhythmia) and slower under stable conditions, mimicking human diagnostic behavior. Where $H_{\omega }$ is a gated update function (Equations 24, 25):

$$s\left(t\right)=\left(1-\alpha_{t}\right)\cdot s\left(t-1\right)+\alpha_{t}\cdot softmax\left(W_{s}\hat{z}\left(t\right)+b_{s}\right),$$ (24)

$$\alpha_{t}=sigmoid\;\left(W_{\alpha }\left[U\left(t\right),\delta \left(t\right)\right]+b_{\alpha }\right),$$ (25)

where δ(t) denotes the rate of latent change $\|\hat{z}\left(t\right)-\hat{z}\left(t-1\right){\|}_{2}$ and $W_{s},W_{\alpha }$ are learned matrices. To model temporal salience, I assign a dynamic attention weight η(t) (Equation 26):

$$\eta \left(t\right)=softmax\;\left(v^{⊤}\tanh\left(W_{\eta }\left[\hat{z}\left(t\right),c\left(t\right)\right]+b_{\eta }\right)\right),$$ (26)

which modulates the contribution of each timestep to the global inference state (Equation 27):

$$S_{\text{agg}}=\overset{T}{\underset{t=1}{\sum }}\eta \left(t\right)\cdot s\left(t\right).$$ (27)

#### 3.4.2 Uncertainty modulation framework

I apply a decision gating module to map the aggregated state to a final diagnostic outcome (Equations 28, 29):

$${\hat{y}}_{\text{final}}=G_{\xi }\left(S_{\text{agg}},U_{\text{agg}},m\right)={argmax}_{j}\;\pi_{j},$$ (28)

$$\pi_{j}=\frac{\exp\left(\gamma \cdot \left[S_{\text{agg}}\left(j\right)-\lambda \cdot U_{\text{agg}}\left(j\right)+\nu \cdot m_{j}\right]\right)}{\sum_{k}\exp\left(\cdot \right)},$$ (29)

where $U_{\text{agg}}=\frac{1}{T}\sum_{t}U\left(t\right)$ is average uncertainty and $m_{j}$ encodes medical risk priors for class j . To encourage calibrated and interpretable reasoning, I introduce an entropy regularized trust gate (Equations 30, 31):

$$T\left(t\right)=sigmoid\;\left(W_{T}\hat{z}\left(t\right)+b_{T}\right),$$ (30)

$$L_{\text{trust}}=\frac{1}{T}\overset{T}{\underset{t=1}{\sum }}T\left(t\right)\cdot KL\left(\hat{y}\left(t\right)\|{\hat{y}}_{\text{final}}\right).$$ (31)

#### 3.4.3 Hierarchical decision logic

AHSIM also supports hierarchical health reasoning. Let $Y_{\text{coarse}}$ and $Y_{\text{fine}}$ be coarse-grained and fine-grained diagnostic categories, and define mapping $\pi :Y_{\text{fine}}\to Y_{\text{coarse}}$ . The hierarchical likelihood is (Equations 32, 33):

$${\hat{y}}_{\text{coarse}}\left(i\right)=\underset{j\in \pi^{-1}\left(i\right)}{\sum }{\hat{y}}_{\text{final}}\left(j\right),$$ (32)

$$L_{\text{hier}}=CE\left(y_{\text{coarse}},{\hat{y}}_{\text{coarse}}\right).$$ (33)

To accommodate deployment-specific tolerances, I design an adaptive decision thresholding (Equations 34, 35):

$$\theta \left(t\right)=\theta_{0}+\epsilon \cdot U\left(t\right)+\zeta \cdot Var\left[\hat{z}\left(t\right)\right],$$ (34)

$${\hat{y}}_{\text{output}}\left(t\right)=\left\{\begin{array}{cc} {\hat{y}}_{\text{final}},\; & \text{if }\max_{j}\pi_{j}\geq \theta \left(t\right)\\ defer,\; & \text{otherwise} \end{array}\right.$$ (35)

The overall optimization combines multi-resolution diagnostic objectives (Equation 36):

$$L_{\text{AHSIM}}=L_{\text{CE}}\left({\hat{y}}_{\text{final}},y\right)+\lambda_{1}L_{\text{trust}}+\lambda_{2}L_{\text{hier}}.$$ (36)

## 4 Experimental setup

### 4.1 Dataset

Biosensor Signal Analysis Dataset Sutar et al. (2025) comprises a diverse range of physiological signals collected from wearable biosensors, including electrodermal activity (EDA), skin temperature, and accelerometer data. These multimodal data streams were gathered from a heterogeneous population under different activity conditions such as resting, walking, and stress-induced tasks. Each data sample is time-stamped, enabling fine-grained temporal analysis. The dataset emphasizes variability in sensor placement, motion artifacts, and real-world conditions to challenge signal processing robustness. Preprocessing steps include low-pass filtering and normalization, ensuring consistency across recordings. The dataset serves as a benchmark for evaluating machine learning algorithms in biosignal classification, anomaly detection, and multimodal fusion tasks, particularly under non-ideal recording environments. Real-Time Health Monitoring Dataset Liu and Du (2024) contains continuous recordings of physiological parameters including heart rate, oxygen saturation, and respiration rate, captured from real-time remote monitoring systems deployed in outpatient settings. It includes both baseline and abnormal health states, annotated by clinical professionals. Each record spans several hours, enabling exploration of long-term health trends and real-time anomaly detection algorithms. The dataset includes contextual metadata such as age, gender, and clinical history, enabling patient-specific modeling. It is suitable for research in predictive diagnostics, real-time monitoring models, and wearable system calibration. Biomedical Sensor Data Collection Suthar et al. (2023) offers synchronized multimodal recordings from multiple biosensors including EEG, EMG, ECG, and PPG signals. The dataset targets laboratory-controlled experimental sessions with varied stimuli to elicit distinct physiological responses. High-resolution annotations mark significant events, enabling the study of reaction-based models and supervised learning approaches. It is tailored for evaluating the temporal resolution of biosignal analytics and exploring signal interdependencies. This dataset provides an ideal ground for sensor fusion studies, as each subject was recorded under identical experimental protocols. Deep Learning Biosensing Dataset Geng et al. (2022) was designed specifically for training deep neural networks on raw sensor inputs. It includes a large volume of labeled biosensor data collected from wearable systems across multiple sessions and subjects. Data types include ECG, body temperature, and galvanic skin response, with synchronized labels for health conditions such as fatigue, dehydration, and stress. The dataset provides extensive training-validation-test splits, data augmentation protocols, and baseline benchmarks, fostering reproducibility in deep learning model development. It also supports unsupervised and semi-supervised learning paradigms, addressing the challenge of limited labeled biomedical data.

The datasets collectively span a wide range of biosignals, including ECG, EEG, EMG, PPG, EDA, skin temperature, respiration rate, and accelerometry. To maintain clinical relevance and consistency across datasets, preprocessing steps were applied uniformly. These included bandpass filtering (0.5–40 Hz for ECG), z-score normalization across temporal windows, wavelet-based motion artifact removal, and synchronization of multimodal inputs at 100 Hz. Missing values were interpolated or masked during training to simulate real-world conditions, ensuring robustness under incomplete data.

### 4.2 Experimental details

All experiments were conducted using PyTorch on NVIDIA A100 GPUs with 40 GB memory. The training and evaluation pipelines were implemented using a modular architecture to ensure reproducibility and scalability. For all datasets, signals were first resampled to a unified frequency of 100 Hz, and each segment was normalized using z-score normalization across the temporal axis. A sliding window of 5 s with 50% overlap was applied to construct fixed-length input samples. Signal augmentation techniques, including Gaussian noise injection, random cropping, and temporal warping, were applied during training to improve generalization.

I adopted a deep convolutional architecture inspired by ResNet-18 as the base encoder. The input signals were treated as multi-channel time-series inputs, with each modality occupying one channel. The model comprises four residual blocks followed by global average pooling and a fully connected layer. Batch normalization and ReLU activation were applied after each convolution. To support multi-modal fusion, a modality attention module was incorporated before the final classifier. For sequence-level tasks, a Bi-LSTM layer was appended after feature extraction to capture temporal dependencies. For classification, a softmax layer was used, and for regression tasks, a linear layer predicted the physiological value.

The models were trained using the Adam optimizer with an initial learning rate of 1e-3, which was decayed by a factor of 0.1 every 10 epochs. A batch size of 64 was used across all experiments. Early stopping with a patience of 20 epochs was employed to prevent overfitting. Cross-entropy loss was used for classification tasks, while mean squared error was adopted for regression problems. For datasets with class imbalance, I applied weighted loss based on inverse class frequency. Each experiment was repeated five times with different random seeds, and I reported the average and standard deviation of all metrics.

To improve the transparency, reproducibility, and replicability of our experimental procedures, I provide a detailed summary of all key parameter settings in Table 1. This includes information on the optimizer choice, learning rate scheduling, batch size, window segmentation, and hardware configurations. In particular, all experiments were conducted using the Adam optimizer with an initial learning rate of 1e-3, and early stopping was employed to prevent overfitting. I also specify architectural components such as the backbone ResNet-18 variant, Bi-LSTM sequence modeling layers, and modality attention mechanisms used for multimodal fusion. These parameters were consistently applied across all datasets unless otherwise noted. By disclosing these details, I aim to facilitate reproducibility and support future comparative studies on biosensing data interpretation frameworks.

**TABLE 1:** Summary of key experimental parameters used in the study.

Parameter	Value/setting
Optimizer	Adam
Initial learning rate	1e-3 (decayed by 0.1 every 10 epochs)
Batch size	64
Window length	5 s
Window overlap	50%
Input sampling frequency	100 Hz
Epochs	Up to 100 (early stopping with patience = 20)
Loss function (classification)	Cross entropy
Loss function (regression)	Mean squared error (MSE)
Network backbone	ResNet-18 variant
Sequence modeling layer	Bi-LSTM with hidden size = 128
Modality attention	Yes (applied before final classifier)
Regularization	Dropout (rate = 0.3), weight decay = 1e-5
Hardware	NVIDIA A100 GPU, 40 GB

For evaluation, I employed accuracy, precision, recall, F1-score, and area under the ROC curve (AUC) for classification. For regression tasks, Pearson correlation coefficient and RMSE were reported. To ensure fairness, all baseline models were re-implemented under identical preprocessing and training conditions. In cases where published code was available, I directly adopted the official implementations and hyperparameters. Otherwise, I followed the best practices as described in the respective papers and tuned parameters through grid search.

Ablation studies were conducted to analyze the contributions of different components including modality attention, sequence modeling, and signal augmentation. I also performed leave-one-subject-out cross-validation (LOSO) for inter-subject generalization analysis and 5-fold cross-validation for intra-subject experiments. All experimental protocols and model checkpoints are released for reproducibility. The overall experimental setup adheres closely to standards adopted in top-tier conferences such as NeurIPS, ICLR, and AAAI, ensuring scientific rigor and comparability with state-of-the-art benchmarks.

### 4.3 Comparison with SOTA methods

Table 2 presents the performance comparison of our proposed method with several state-of-the-art (SOTA) baselines, including TextCNN, BERT, RoBERTa, ALBERT, DistilBERT, and T5, on the Biosensor Signal Analysis and Real-Time Health Monitoring Datasets. Our method achieves the best performance across all evaluation metrics, with a notable accuracy of 92.48% and 91.87% on the two datasets respectively, surpassing the strongest baseline (RoBERTa) by approximately 2.34% and 3.87%. The F1 score, recall, and AUC also reflect consistent gains, with improvements ranging from 1.8% to 3.5% over the second-best performing model. These results indicate that our model not only captures high-level semantic representations effectively but also retains critical temporal and multimodal dependencies that transformer-based models such as BERT and T5 tend to overlook in biosignal domains. While transformer-based architectures generally excel at textual representation learning, their direct application to continuous biomedical signals appears suboptimal due to lack of inductive bias for temporal signal patterns and noise-resilience, especially under conditions of signal artifacts and sensor drift. Additionally, lighter models such as DistilBERT suffer from significant performance drops, reinforcing that simple model compression leads to loss of nuanced physiological information necessary for reliable predictions in biosensor analysis.

**TABLE 2:** Comparison of ours with SOTA methods on biosensor signal analysis and real-time health monitoring datasets.

Model	Biosensor signal analysis dataset				Real-time health monitoring dataset			
	Accuracy	Recall	F1 score	AUC	Accuracy	Recall	F1 score	AUC
TextCNN Duarte and Berton (2023)	87.52 ± 0.03	85.10 ± 0.02	84.67 ± 0.03	88.34 ± 0.02	85.24 ± 0.02	84.76 ± 0.02	83.90 ± 0.02	86.11 ± 0.03
BERT Soni et al. (2022)	89.63 ± 0.02	87.40 ± 0.02	88.12 ± 0.02	90.08 ± 0.02	87.35 ± 0.03	85.97 ± 0.02	86.74 ± 0.03	88.65 ± 0.02
RoBERTa Min et al. (2021)	90.14 ± 0.02	88.33 ± 0.03	87.97 ± 0.02	91.20 ± 0.02	88. ± 0.02	87.01 ± 0.02	86.58 ± 0.02	89.30 ± 0.02
ALBERT Minaee et al. (2020)	88.05 ± 0.02	86.12 ± 0.02	85.79 ± 0.02	89.41 ± 0.03	86.73 ± 0.02	85.90 ± 0.03	84.55 ± 0.03	87.88 ± 0.03
DistilBERT Kowsari et al. (2019)	86.40 ± 0.02	84.29 ± 0.02	83.95 ± 0.02	87.13 ± 0.02	85.61 ± 0.03	83.47 ± 0.02	82.93 ± 0.02	86.04 ± 0.02
T5 Wang et al. (2018)	89.17 ± 0.03	87.10 ± 0.03	86.40 ± 0.03	90.03 ± 0.03	88.20 ± 0.02	86.35 ± 0.03	85.77 ± 0.02	88.44 ± 0.02
Ours	**92.48 ± 0.02**	**90.81 ± 0.02**	**90.30 ± 0.03**	**93.65 ± 0.02**	**91.87 ± 0.02**	**89.72 ± 0.02**	**89.11 ± 0.03**	**92.10 ± 0.03**

The bolded values represent the optimal values.

Table 3 further evaluates the same set of models on the Biomedical Sensor Data Collection and Deep Learning Biosensing Datasets. Once again, our approach consistently outperforms all SOTA models across every metric. Specifically, our model achieves an accuracy of 91.88% and 90.97%, reflecting a significant edge of over 2%–3% compared to the next-best method, T5. Our method’s superiority is particularly prominent in AUC scores, registering 93.01 and 91.60 respectively, which underscores our system’s strong capability in distinguishing subtle health-related classes under varying distributions and session-level variability. The robustness of our model arises from its integrated modality attention mechanism, which dynamically weighs each biosignal modality, allowing it to downplay noisy or uninformative channels. In contrast, traditional NLP-based methods such as BERT and RoBERTa lack explicit support for channel-wise feature fusion, limiting their flexibility when dealing with heterogeneous sensor inputs. Furthermore, our framework incorporates sequential modeling layers such as Bi-LSTM, which captures temporal evolution of physiological signals more effectively than position-encoded transformers that assume discrete token boundaries. This is crucial in scenarios such as reaction-based EMG or fatigue-related ECG patterns, where signal latency and transitional phases hold diagnostic importance. Therefore, while the performance gap may appear moderate in numeric terms, it represents substantial real-world gains in biosignal monitoring, where even minor improvements in recall or AUC can translate into reduced false alarms or improved early detection rates.

**TABLE 3:** Comparison of ours with SOTA methods on biomedical sensor data collection and deep learning biosensing datasets.

Model	Biomedical sensor data collection				Deep learning biosensing dataset			
	Accuracy	Recall	F1 score	AUC	Accuracy	Recall	F1 score	AUC
TextCNN Duarte and Berton (2023)	85.42 ± 0.02	83.17 ± 0.02	84.80 ± 0.02	86.95 ± 0.03	84.73 ± 0.03	82.24 ± 0.02	81.88 ± 0.02	85.31 ± 0.02
BERT Soni et al. (2022)	88.61 ± 0.02	86.94 ± 0.03	86.11 ± 0.03	89.42 ± 0.02	87.89 ± 0.02	85.17 ± 0.02	86.66 ± 0.02	88.27 ± 0.03
RoBERTa Min et al. (2021)	87.50 ± 0.03	85.28 ± 0.02	84.95 ± 0.02	88.90 ± 0.02	86.45 ± 0.02	84.71 ± 0.02	83.99 ± 0.03	87.83 ± 0.02
ALBERT Minaee et al. (2020)	86.33 ± 0.02	84.42 ± 0.02	83.60 ± 0.02	87.05 ± 0.02	85.10 ± 0.02	83.77 ± 0.02	82.85 ± 0.02	86.24 ± 0.02
DistilBERT Kowsari et al. (2019)	84.90 ± 0.03	82.65 ± 0.02	82.10 ± 0.03	85.72 ± 0.03	83.98 ± 0.02	82.44 ± 0.03	80.66 ± 0.02	84.89 ± 0.02
T5 Wang et al. (2018)	89.05 ± 0.02	87.33 ± 0.02	86.74 ± 0.02	89.70 ± 0.03	88.42 ± 0.02	86.59 ± 0.02	86.17 ± 0.02	88.92 ± 0.03
Ours	**91.88 ± 0.02**	**90.41 ± 0.03**	**89.76 ± 0.03**	**93.01 ± 0.02**	**90.97 ± 0.02**	**89.50 ± 0.02**	**88.93 ± 0.03**	**91.60 ± 0.02**

The bolded values represent the optimal values.

Across all four datasets, the performance margins suggest that our approach generalizes well across different biosensing scenarios—ranging from stress detection to long-term health monitoring. These gains can be attributed to several design factors: (1) the incorporation of temporal convolution and sequence modeling, which captures both local and global signal transitions; (2) the modality-specific attention mechanism, which enhances feature fusion across diverse signal types; and (3) extensive use of data augmentation, which regularizes the model and improves robustness to real-world noise. While models like T5 and RoBERTa show strong baseline performance due to their pretraining on large-scale corpora, their adaptation to biosignal domains is limited by their reliance on discrete token structures and lack of alignment with the continuous and often noisy nature of sensor data. Our model, being specifically tailored for the biosensing context, demonstrates the importance of designing architectures that are sensitive to both temporal and physiological signal characteristics. Moreover, the consistent superiority across both inter-subject (Real-Time Health Monitoring) and intra-session (Deep Learning Biosensing) datasets suggests that our method is both robust and scalable. This highlights its potential for real-world deployment in healthcare applications requiring high reliability and minimal latency, and further supports the utility of incorporating physiological priors into deep learning architectures for biosignal analysis.

### 4.4 Ablation study

To evaluate the contributions of the proposed components in our PhysioGraph Inference Network (PGIN), I conducted an ablation study across four datasets. The results are presented in Table 4 and 5. Three ablated variants were analyzed: (1) w/o Temporal Graph Convolution, which removes the temporal graph convolution layer responsible for capturing inter-sensor dependencies; (2) w/o Latent Variable Modeling, which excludes the latent variable modeling framework for temporal dynamics; and (3) w/o Uncertainty-Aware Decision Heads, which omits the uncertainty-aware decision mechanism. The complete model, referred to as Ours, includes all components and serves as the baseline for comparison.

**TABLE 4:** Ablation study results on ours model across biosensor signal analysis and real-time health monitoring datasets.

Model	Biosensor signal analysis dataset				Real-time health monitoring dataset			
	Accuracy	Recall	F1 score	AUC	Accuracy	Recall	F1 score	AUC
w/o temporal graph convolution	89.76 ± 0.03	87.92 ± 0.02	87.01 ± 0.03	90.45 ± 0.02	88.34 ± 0.03	86.78 ± 0.02	85.92 ± 0.03	88.91 ± 0.02
w/o latent variable modeling	90.54 ± 0.02	88.67 ± 0.03	87.89 ± 0.02	91.12 ± 0.03	89.12 ± 0.02	87.45 ± 0.03	86.78 ± 0.02	89.54 ± 0.03
w/o uncertainty-aware decision heads	91.02 ± 0.03	89.34 ± 0.02	88.56 ± 0.03	91.87 ± 0.02	89.78 ± 0.02	88.12 ± 0.03	87.45 ± 0.02	90.23 ± 0.03
Ours	**92.48 ± 0.02**	**90.81 ± 0.02**	**90.30 ± 0.03**	**93.65 ± 0.02**	**91.87 ± 0.02**	**89.72 ± 0.02**	**89.11 ± 0.03**	**92.10 ± 0.03**

The bolded values represent the optimal values.

**TABLE 5:** Ablation study results on ours model across biomedical sensor data collection and deep learning biosensing datasets.

Model	Biomedical sensor data collection				Deep learning biosensing dataset			
	Accuracy	Recall	F1 score	AUC	Accuracy	Recall	F1 score	AUC
w/o temporal graph convolution	89.45 ± 0.03	87.34 ± 0.02	86.78 ± 0.03	90.12 ± 0.02	88.67 ± 0.03	86.89 ± 0.02	86.01 ± 0.03	89.34 ± 0.02
w/o latent variable modeling	90.12 ± 0.02	88.45 ± 0.03	87.56 ± 0.02	90.89 ± 0.03	89.23 ± 0.02	87.78 ± 0.03	87.01 ± 0.02	89.89 ± 0.03
w/o uncertainty-aware decision heads	90.78 ± 0.03	89.12 ± 0.02	88.34 ± 0.03	91.45 ± 0.02	89.89 ± 0.02	88.34 ± 0.03	87.67 ± 0.02	90.45 ± 0.03
Ours	**91.88 ± 0.02**	**90.41 ± 0.03**	**89.76 ± 0.03**	**93.01 ± 0.02**	**90.97 ± 0.02**	**89.50 ± 0.02**	**88.93 ± 0.03**	**91.60 ± 0.02**

The bolded values represent the optimal values.

The removal of the temporal graph convolution layer (w/o Temporal Graph Convolution) resulted in the most significant performance degradation across all datasets. For example, on the Biosensor Signal Analysis Dataset, accuracy decreased from 92.48% to 89.76%, and the F1 score dropped by over 3%. This indicates the importance of modeling inter-sensor dependencies for accurate representation learning. Excluding the latent variable modeling framework (w/o Latent Variable Modeling) led to a moderate reduction in performance, particularly in recall and F1 scores on datasets with strong temporal dependencies, such as the Biomedical Sensor Data Collection. This highlights the role of latent dynamics in capturing temporal variations. The absence of the uncertainty-aware decision heads (w/o Uncertainty-Aware Decision Heads) showed a smaller but consistent impact, particularly in terms of reduced robustness and increased variance across multiple runs. This suggests that the uncertainty-aware mechanism enhances the model’s reliability under noisy and heterogeneous conditions.

These results validate the architectural choices in the PhysioGraph Inference Network. The temporal graph convolution layer effectively captures inter-sensor relationships, the latent variable modeling framework enhances temporal dynamics, and the uncertainty-aware decision heads improve robustness and generalization. The full model consistently achieves superior performance across all metrics and datasets, demonstrating the synergistic benefits of integrating these components.

### 4.5 Comparison with additional baseline systems

To ensure a fair and comprehensive evaluation of our proposed method, I conducted experiments comparing its performance with several conventional and state-of-the-art baseline models. The selected baselines include shallow neural networks (TextCNN), recurrent architectures (BiLSTM), and transformer-based models (BERT, RoBERTa, and T5). All models were trained under the same preprocessing pipeline, data splits, and evaluation metrics to ensure consistency. Hyperparameters for each baseline were either taken from published implementations or tuned using grid search to reflect optimal performance. Table 6 summarizes the results on two representative datasets: Biosensor Signal Analysis and Biomedical Sensor Data Collection. As shown, our proposed model consistently outperforms all baselines across multiple metrics, particularly in diagnostic accuracy and AUC. This reinforces the utility of incorporating physiological priors, temporal graph reasoning, and uncertainty-aware inference in biosensing diagnostics.

**TABLE 6:** Comparison with additional baseline methods.

Model	Accuracy	Recall	F1 score	AUC
TextCNN	87.52%	85.10%	84.67%	88.34%
BiLSTM	88.40%	86.79%	86.01%	89.15%
BERT	89.63%	87.40%	88.12%	90.08%
RoBERTa	90.14%	88.33%	87.97%	91.20%
T5	89.17%	87.10%	86.40%	90.03%
Ours	**92.48%**	**90.81%**	**90.30%**	**93.65%**

The bolded values represent the optimal values.

### 4.6 Robustness under noisy or missing inputs

To further validate the robustness of the proposed PGIN model, we conducted a series of controlled degradation experiments to simulate real-world sensor corruption. Specifically, two types of perturbations were introduced: (1) additive Gaussian noise with zero mean and 0.1 standard deviation applied to randomly selected sensor channels, and (2) random masking of 10%, 20%, and 30% of sensor channels during inference to mimic sensor dropout or transmission loss.

Table 7 presents the performance of PGIN under varying levels of corruption on the Biosensor Signal Analysis Dataset. Compared to RoBERTa and BiLSTM baselines, PGIN exhibits significantly more stable accuracy, F1 score, and AUC values across all corruption levels. This robustness is attributed to the model’s temporal graph regularization term and the masked dropout strategy employed during training, which jointly enforce smooth and plausible inter-sensor dependency representations. The results confirm that our model maintains interpretability and predictive reliability even under noisy or incomplete data conditions.

**TABLE 7:** Robustness evaluation under sensor noise and missing data (biosensor signal analysis dataset).

Model	Setting	Accuracy	F1 score	AUC
PGIN (ours)	10% corruption	91.84	89.96	92.90
	20% corruption	90.43	88.47	91.32
	30% corruption	88.92	86.65	89.71
RoBERTa	10% corruption	88.51	86.34	89.42
	20% corruption	86.32	83.90	87.25
	30% corruption	84.25	81.60	85.14
BiLSTM	10% corruption	87.32	84.80	88.13
	20% corruption	85.27	82.51	86.04
	30% corruption	83.90	80.35	83.80

### 4.7 Clinician interpretability evaluation

In particular, we found that the latent trajectory visualizations provided a valuable temporal context for understanding patient states. For example, clinicians noted that sharp changes in the trajectory often coincided with labeled events such as onset of arrhythmia or stress episodes, allowing them to retrospectively validate the physiological plausibility of the model’s internal reasoning. Several participants commented that the shape and smoothness of the latent trajectory over time helped them distinguish between stable and deteriorating patient conditions. This suggests that the learned latent space is not only algorithmically informative, but also aligned with clinical intuitions. The ability to cross-reference latent states with observable biosignal dynamics enhances the transparency and trustworthiness of the diagnostic process. This supports the use of such trajectories as verifiable and interpretable representations in real-world medical practice.

To evaluate whether the interpretability of our model translates into clinically actionable insights, we conducted a human-in-the-loop usability study involving six board-certified clinicians from cardiology and critical care departments. Each participant was presented with 50 biosignal cases sampled from the Real-Time Health Monitoring Dataset and asked to perform diagnostic classification under two conditions: (1) using only the raw model predictions, and (2) using model predictions augmented with interpretability cues such as attention heatmaps, uncertainty scores, and latent physiological trajectories.

The results are summarized in Table 8. Clinicians achieved significantly higher diagnostic accuracy and expressed greater confidence and trust when interpretability outputs were available. Specifically, average diagnostic accuracy increased by 12.5%, while perceived trust in the AI system improved by over 40%. Additionally, decision-making time slightly decreased, indicating that interpretability support also improves efficiency. Qualitative feedback highlighted the value of attention maps and latent state visualizations in understanding anomalous biosignal patterns, especially in ambiguous cases. These findings indicate that the interpretability features of our framework go beyond analytical visualization and serve as meaningful tools that support clinical reasoning and foster trust in AI-assisted diagnostics.

**TABLE 8:** Clinician usability study comparing model-only vs. model + interpretability outputs (N = 6 clinicians).

Metric	Model only	Model + interpretability	Relative change
Diagnostic accuracy (%)	74.2	83.5	+12.5%
Confidence score (1–5 scale)	3.1	4.2	+35.5%
Trust in AI decision (1–5 scale)	2.9	4.1	+41.4%
Decision time (sec/sample)	14.3	12.8	−10.5%

## 5 Conclusions and future work

This work proposes an interpretable and uncertainty-aware deep learning framework for biosensing-based health diagnostics. By introducing the PhysioGraph Inference Network (PGIN), we enable dynamic modeling of physiological signal relationships using graph-based structures grounded in clinical priors. The Adaptive Health State Inference Mechanism (AHSIM) further enhances the framework’s decision-making process by integrating temporal memory, uncertainty quantification, and diagnostic granularity adjustment. Extensive evaluations across four diverse datasets demonstrate that our approach outperforms existing baselines in terms of accuracy, robustness, and explainability, making it suitable for deployment in both clinical and wearable settings.

Beyond algorithmic performance, the model’s interpretability components are designed to generate clinically meaningful outputs. Latent physiological trajectories produced by the model can be visualized and cross-referenced with biosignal events, offering diagnostic cues that align with expert knowledge. Attention mechanisms and modality-aware encoders allow clinicians to trace decision factors back to source signals, enabling hybrid human-AI workflows that support transparency and reduce diagnostic risk.

Despite these contributions, the framework has several limitations. The symbolic abstraction of latent states may not capture all nuances in atypical physiological conditions. Additionally, while the model is adaptable to different sensor types, further validation is needed across larger, more heterogeneous patient populations and devices. Future work will focus on integrating structured physiological knowledge graphs, improving edge deployment efficiency, and conducting human-in-the-loop studies to better assess clinical utility in real-world practice.

## Data Availability

The original contributions presented in the study are included in the article/supplementary material, further inquiries can be directed to the corresponding author.
